# Correlation Analysis of Variables From the Atherosclerosis Risk in Communities Study

**DOI:** 10.3389/fphar.2022.883433

**Published:** 2022-07-11

**Authors:** Meisha Mandal, Josh Levy, Cataia Ives, Stephen Hwang, Yi-Hui Zhou, Alison Motsinger-Reif, Huaqin Pan, Wayne Huggins, Carol Hamilton, Fred Wright, Stephen Edwards

**Affiliations:** ^1^ GenOmics, Bioinformatics, and Translational Research Center, RTI International, Research Triangle Park, NC, United States.; ^2^ Levy Informatics, Chapel Hill, NC, United States; ^3^ Department of Statistics, North Carolina State University, Raleigh, NC, United States; ^4^ Bioinformatics Research Center and Department of Biological Sciences, North Carolina State University, Raleigh, NC, United States; ^5^ Biostatistics and Computational Biology Branch, National Institute of Environmental Health Sciences, Durham, NC, United States

**Keywords:** cluster analysis, systems biology, meta-analysis as topic, ARIC, cardiovascular disease

## Abstract

The need to test chemicals in a timely and cost-effective manner has driven the development of new alternative methods (NAMs) that utilize in silico and *in vitro* approaches for toxicity prediction. There is a wealth of existing data from human studies that can aid in understanding the ability of NAMs to support chemical safety assessment. This study aims to streamline the integration of data from existing human cohorts by programmatically identifying related variables within each study. Study variables from the Atherosclerosis Risk in Communities (ARIC) study were clustered based on their correlation within the study. The quality of the clusters was evaluated via a combination of manual review and natural language processing (NLP). We identified 391 clusters including 3,285 variables. Manual review of the clusters containing more than one variable determined that human reviewers considered 95% of the clusters related to some degree. To evaluate potential bias in the human reviewers, clusters were also scored via NLP, which showed a high concordance with the human classification. Clusters were further consolidated into cluster groups using the Louvain community finding algorithm. Manual review of the cluster groups confirmed that clusters within a group were more related than clusters from different groups. Our data-driven approach can facilitate data harmonization and curation efforts by providing human annotators with groups of related variables reflecting the themes present in the data. Reviewing groups of related variables should increase efficiency of the human review, and the number of variables reviewed can be reduced by focusing curator attention on variable groups whose theme is relevant for the topic being studied.

## 1 Introduction

The past 2 decades have seen a transition away from toxicity testing using laboratory animals to new alternative methods (NAMs) that rely on computational predictions or *in vitro* assays. These new methods have the advantage of being able to screen many more chemicals at a highly reduced cost while simultaneously reducing animal suffering. When being used to support human health risk assessment, these approaches can have the added benefit of using human cells or computational models built using human parameters to avoid the need for species extrapolation (Krewski et al., 2010). However, these advantages are lost if the NAMs are evaluated using the traditional animal toxicity predictions as the gold standard (Interagency Coordinating Committee on the Validation of Alternative Methods, 2018; Piersma et al., 2018; Ly Pham et al., 2020). There is a need to pivot towards an evaluation paradigm that uses data on adverse outcomes in humans for evaluating toxicity predictions in order to fully realize the vision of toxicity testing in the twenty first century (Krewski et al., 2010).

In 2010, the adverse outcome pathway (AOP) was proposed as a framework for interpreting the outputs from NAMs (Ankley et al., 2010). With support from the Organisation for Economic Co-operation and Development (OECD), this framework has supported a wide array of applications for integrating and translating toxicity predictions from NAMs (Cote et al., 2016; Schultz et al., 2016; Wittwehr et al., 2017; Ankley and Edwards, 2018a; Watford et al., 2019a; Spinu et al., 2020; Goyak and Lewis, 2021). While most applications to date have focused on integrating data from *in vitro* assays and laboratory animals, the process for incorporating human data would be equivalent. By incorporating the human data into the AOP framework, we can then use the human data as our gold standard. This avoids potential complications that arise from using data from laboratory animals as the gold standard when those endpoints are an imperfect indicator of the actual outcome of interest in the target population.

Over the last few decades, vast amounts of human data have been collected for clinical and research purposes. Resources such as the database of Genotypes and Phenotypes (dbGaP) (Mailman et al., 2007), United Kingdom Biobank (Sudlow et al., 2015), All of Us (All of Us Research Program Investigators et al., 2019), and CHEAR/HHEAR databases (Balshaw et al., 2017) have been developed to archive and facilitate sharing/distribution of these data. The combination of large cohorts from these studies offers the potential of data pooling and meta-analysis possessing sizable statistical power. Meta-analysis and pooling of data from multiple studies create value well beyond that of the original research by increasing data reproducibility and robustness. Additionally, data pooling increases the sample size, which has a multitude of benefits including increased statistical power and potential to support increasingly complex analysis models. However, this requires significant data harmonization, which can be labor intensive. Curation of data to adhere to FAIR principles (Findable, Accessible, Interoperable, Reusable) (Wilkinson et al., 2016) is also a labor-intensive process which requires each variable to be reviewed and curated.

Previous harmonization efforts, such as the mapping of PhenX variables to the dbGaP and LOINC vocabulary (Pan et al., 2012), the establishment of a shared measurement framework for ECHO (Blackwell et al., 2018), the development of the Semantic Data Dictionary (Rashid et al., 2020), the phenotype harmonization system developed by the TOPMed program (Stilp et al., 2021), and the HHEAR resource model (Viet et al., 2021) have been successful and increased the potential for cross-study and transdisciplinary analysis. However, as acknowledged by the authors of many of these studies, manual data harmonization is a laborious, time-consuming, and not easily scalable process. As this is a problem common to many data harmonization and curation efforts, developing an automated method to assist in this process would reduce the amount of labor involved and thereby encourage researchers to undertake these valuable efforts. For example, consider the impact of automation on the Cure Sickle Cell Initiative (CureSCi) MetaData Catalog (MDC) (Pan et al., 2021). The CureSCi MDC is an effort to make Sickle Cell Disease (SCD) study datasets more Findable in accordance with FAIR principles. The development of the CureSCi MDC involved manual curation of data into a three-tiered conceptual framework consisting of category, subcategory, and data elements. Having variables programmatically grouped into multiple tiers of related variables would significantly expedite this manual curation process.

The objective of this study is to develop a data-driven method to cluster related variables and further assemble those clusters into higher-order groups and topics, which can support data harmonization and curation efforts. This would provide a tiered organizational structure analogous to the one used in development of the CureSCi MDC with variables serving as potential data elements, clusters as potential subcategories, and cluster groups as potential categories, which could then be used as the starting point for the manual curation. To accomplish this, we performed a large-scale correlation analysis and arranged variables into a multi-tier organizational structure consisting of variables, variable clusters, cluster groups and topics. Our analysis focused on assembling the variables from a single study to demonstrate that biologically meaningful groups of variables can be assembled programmatically. The next logical step would be to apply this method across multiple studies and demonstrate the value for supporting data harmonization.

## 2 Materials and Methods

### 2.1 ARIC Variable Correlation, Filtering, and Clustering

#### 2.1.1 Correlation Analysis

The Atherosclerosis Risk in Communities (ARIC) Study (N = 15,792) (The Atherosclerosis Risk in Communities Study, 1989) is a large-scale, prospective study investigating cardiovascular health in African Americans that has been tracking participants since 1987 and is still ongoing. The goal of the ARIC study is to investigate the causes and risk factors of cardiovascular disease (CVD), atherosclerosis, and stroke as well as the connections between cardiovascular and cognitive health. Data from the ARIC study were obtained through the BioLINCC data repository (Giffen et al., 2015).

**FIGURE 1:**
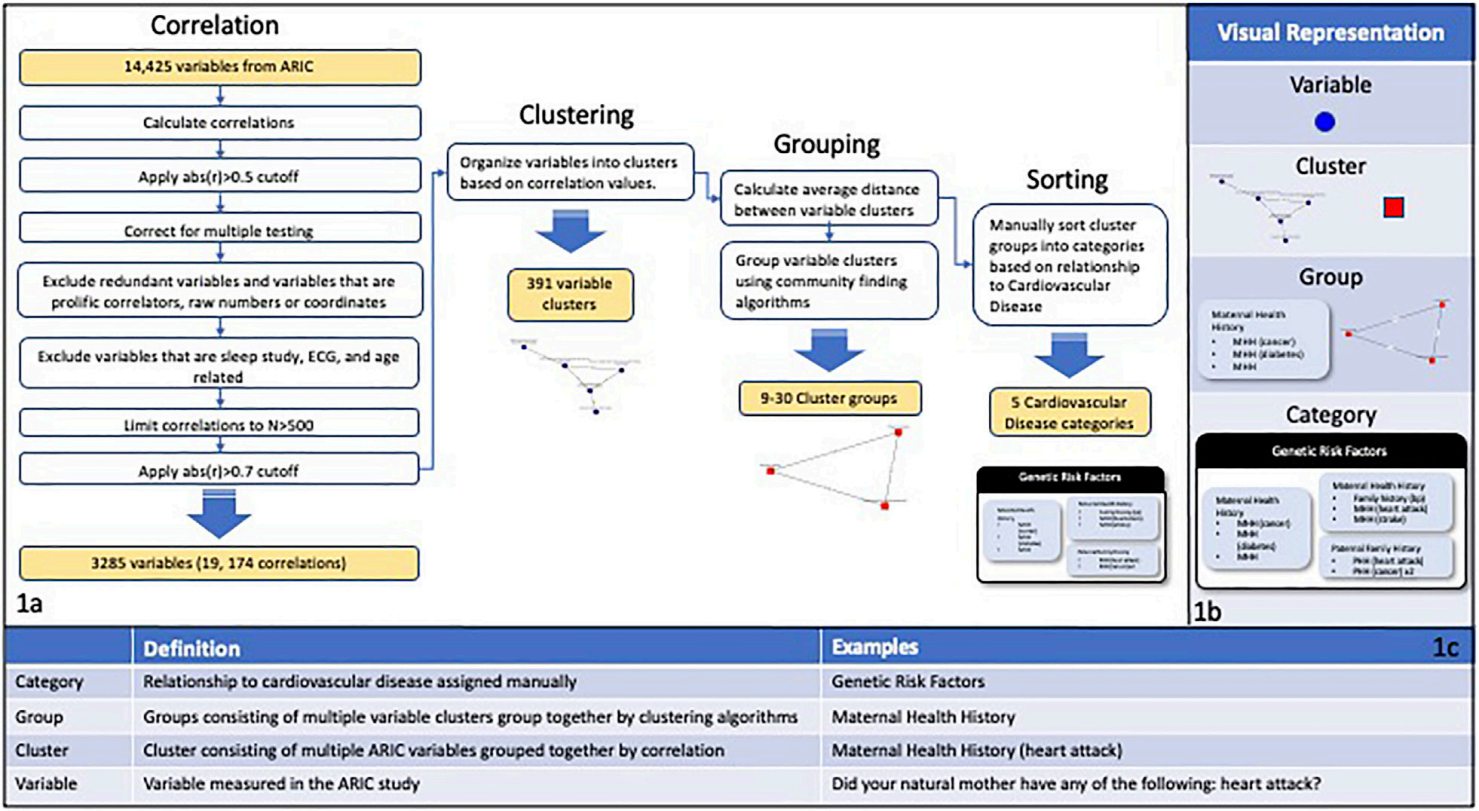
**(A)** Depicts the correlation, filtering, and clustering process applied to the 14,425 variables in the ARIC study. The variable correlations were calculated and then multiple filtering steps were performed including filtering by cutoff and N values, correcting for multiple testing, and excluding specific categories of variables. The variables were organized into clusters of interconnected nodes based on the filtered correlation values, resulting in 391 variable clusters. The average distance between variable clusters was calculated and clusters were grouped using community finding algorithms. The cluster groups were manually sorted into categories based on the main goals of the ARIC study. **(B)** Visual representations of the different levels of organization used in this study. **(C)** A chart showing definitions and examples for the different levels of organization used in this study.

A correlation analysis was done on 14,425 phenotype-associated variables from the ARIC study (Figure 1). For this proof-of-concept study, the Pearson correlation was used for all variables. Future work will evaluate additional correlation methods and goodness of fit tests to account for variables with skewed distributions and non-linear relationships between variables. The analysis included the following: 1) calculating pairwise partial correlations using age and sex as covariates; 2) removing pairs where the absolute value of correlation was less than 0.5 3) merging redundant variables (e.g., equivalent variables measured at different time points); 4) employing a simple empirical Bayes shrinkage model to account for varying effect sizes and to estimate the “true” trait-trait correlations (see Section 2.1.2); 5) removing variables with prolific correlations (mean number of correlations per variable = 164) and age-related variables; 7) limiting correlations to those based on a sample size of at least 500 subjects. We removed variables with prolific correlations, such as age, because they are not useful for extracting meaningful relationships among variables due to their high number of correlations. This process resulted in 19,174 correlations including 3,285 variables. The resulting filtered correlation matrix was converted to a network graph with edge weights between two variables given by the absolute value of the partial correlation between the variables. Clusters of variables were defined by removing edges with a weight of less than 0.7 and identifying the connected subgraphs within the resulting network. This resulted in 391 variable clusters. Clusters corresponding to the connected subgraphs within the network were then evaluated as described below.

#### 2.1.2 An Empirical Bayes Shrinkage Correlation Estimate

A common feature in the data is that correlation estimates for different pairs of variables may be based on substantially different sample sizes, creating difficulties in comparisons across variable pairs. One solution is to use the variability in estimated correlations to devise appropriate shrinkage factors, which would aggressively shrink correlations based on few observations. For the *i*th Pearson correlation *r*_*i*_ based on an observed (non-missing) sample size *n*_*i*_, an estimate of the sampling variance is *v*_i_=(1-*r*_*i*_^2^)^2^/ *n*_*i*_, and *r* and *v* the vectors of these values across all variable pairs. The quantity τ^2^ = max (0,var(*r*)-mean(*v*)) is an estimate of the underlying variance of true correlations ρ, and *μ* = mean(*r*) an estimate of the true average ρ. For each pairwise correlation, the quantity *r*_*shrunk,i*_=(τ^2^/(τ^2^ + *v*_*i*_))(*r*_*i*_-μ)+μ is the best linear predictor (McCulloch and Searle, 2004) for the true correlation ρ_*i*_, shrinking correlations based on small samples more than those based on large samples.

### 2.2 Cluster Evaluation

Clusters were manually evaluated by a team of 3 reviewers and categorized as “exact,” “highly related,” “related,” and “unrelated”. Following the initial review, a single independent reviewer evaluated all clusters and adjusted categories in consultation with the initial reviewers to increase consistency. Ratings were based on the degree of correlation expected among the variables, as determined by the human reviewers (Table 1).

**TABLE 1:** Examples of human and programmatic evaluation of variable clusters. The table includes the relatedness category from manual review (Category), working definition of the category used by reviewers (Definition), examples of types of relationships in the category (General Examples), examples of ARIC variables that fit each relationship type (Study Variables), a cluster identifier (Cluster Number), and calculated relatedness score from the NLP analysis (Score). The scoring process is described in further detail in the methods section. Examples were selected to demonstrate different types of variable relationships that exist among ARIC variables and the associated relatedness category. See Supplemental Table S1 for all clusters.

Category	Definition	General examples	Study variables	Cluster Number	Score
Unrelated	Clusters where a human reviewer would not expect correlation between the variables in the cluster.	Clusters related to a topic, such as MRI exclusion criteria, but are disparate and would not be expected to correlate	“Do you have a cardiac pacemaker or a heart valve prosthesis?” and “Do you have metal fragments in your eyes, brain, or spinal cord?”	269	8.5
			“Enter code and specify brand and form below” and “What kind of fat do you usually use for baking?”	213	7.9
Related	Clusters where the variables would be expected to be correlated but not as highly would be “related”.	Clusters where the variables all relate to the same broad topic, such as history of cardiovascular disease	“Medications which secondarily affect cholesterol,” “Average mean arterial blood pressure,” and “Carotid Distensibility”	1	10.5
		Clusters relating dietary intake of a nutrient and blood level of that nutrient	“In the past year, how often on average did you consume... Dark meat fish, such as salmon, mackerel, swordfish, sardines, bluefish” and “Omega fatty acid W20:5 and W22:6 [g]”	38*3*	11.6
Highly Related	Clusters where a human reviewer would expect a high degree of correlation between the variables.	Clusters where one variable depends on the other	“Ever had emphysema” and “Age emphysema started”	16	35.1
		Clusters where the variables all relate to the same narrow topic such as consumption of alcoholic beverages, or a history of wheezing	“How many drinks of hard liquor do you usually have per week?,” “How many days in a week do you usually drink beer?” and “Alcohol intake [g] per day”	46	17.7
			“[Wheezing]. Ever have to stop for breath when walking at our own pace on the level?” and “[Wheezing]. Ever stop for breath after walking about 100 yards (or after a few minutes) on the level?”	248	40.5
Exact	Clusters where a human reviewer would expect almost complete correlation between the variables.	Clusters with variables that are repeat measurements during the same exam	First, second and third sitting blood pressure measurement at exam 2	58	44.2
		Clusters with variables that ask the same question, potentially in different ways	“I have a fiery temper,” “I am hotheaded,”, and “I am quick tempered”	86	32.2
			“Have you ever been diagnosed by a doctor as having a polyp or noncancerous tumor of the colon or rectum?” and “Has a doctor ever told you that you had adenoma or polyp of the colon (large intestine)?”	175	32.2
		Clusters with variables that are the same measurement at different time points	White blood cell count at exams 3 and white blood cell count at exam 4	226	47.2

Clusters were also scored programmatically for relatedness using Natural language processing (NLP) tools available in the Python Natural Language Toolkit package (Bird et al., 2009). We processed the variable text using lemmatization, customized stop word removal, and regular expression (regex) substitution to increase standardization. We then mapped the processed variables to the MESH (Coletti and Bleich, 2001), STO (Habibi-Koolaee et al., 2021), and SNOMED (Stearns et al., 2001) biomedical ontologies and controlled vocabularies using Bioportal (Whetzel et al., 2011). The mapping included obtaining direct annotations and ancestors up to 3 levels above. Next, we processed the variable text and annotation results using NLP tokenization and stemming to facilitate comparison between variables. This resulted in a list of processed variable terms (variable term list) and a list of processed annotation terms (annotation term list) for each variable. We performed a pairwise comparison of variables, calculating the percentage overlap (number of common words/total number of words) between terms on the variable term list and terms on the annotation term list for each pair of variables. The overall score for a cluster was the mean overlap of variable terms and annotation terms for all variable pairs in the cluster.

### 2.3 Cluster Grouping and Sorting

A graphical representation considering variable clusters as nodes and inter-cluster distances as edges was used to group clusters. The overall distance between two clusters was defined as the mean value of the inter-cluster edges between all variables in the two clusters. When feasible, each cluster was labeled with a common theme, such as “cough” or “maternal health history.”

Variable clusters were then split into cluster groups using community finding algorithms. In order to select the algorithm that best fit our task, we tried several different community-finding algorithms from Python 3.9’s python-louvain package (v0.14) and NetworkX package (v2.4) using default parameters except when it is stated. This includes the Louvain, Asynchronous label-propagation (LPA), Asynchronous Fluid Communities (number of communities = 15), Greedy Modularity Communities, Girvan-Newman, and K-clique (minimum clique size = 3) algorithms. Prior to running the algorithm, the graph edges connecting clusters were pruned based on the edge weights to a threshold of 0.08–0.25 to facilitate the community finding process. Clusters related to retinal exams or sleep studies, as well as Minnesota code data, administrative data, insurance data, specimen collection and processing data, and quality data were excluded from the community finding process as they were unlikely to be informative regarding mechanisms of disease and were prolific correlators due to the nature of the variables. We ultimately selected the Louvain algorithm as our community-finding algorithm because it formed the largest number of highly coherent cluster groups. The final cluster groups in Supplemental Table S2 were defined based on python-louvain’s best_partition () function (default parameters) which uses the Louvain community finding algorithm. Given the proof-of-concept nature of this work, we only tested a limited subset of existing community finding algorithms. Future work could focus on fine-tuning the community finding component of our workflow.

When plotting a single cluster group, edges below a set viewing threshold value ranging from 0.03–0.15 were removed to enhance the plot’s readability (Figure 4). When plotting multiple cluster groups together such as in Figure 5, the threshold used to create the cluster groups (ranging from 0.08–0.25) became the lower-limit for intra-cluster edges and the threshold for inter-cluster edges was set at 0.05 (Figure 5). Edges with weights below the relevant threshold were removed prior to plotting.

Lastly, to summarize the study data, we manually assigned theme-based labels and organized cluster groups into topics based on the ARIC study goals (lifestyle/environmental risk factors, genetic risk factors, medical care, clinical outcomes, and co-morbidities).

## 3 Results

### 3.1 Atherosclerosis Risk in Communities Variable Clusters

Clustering ARIC variables based on the partial correlations resulted in 3,285 variables organized into 391 clusters, referred to as variable clusters, containing between 2 and 634 ARIC variables with 385/391 (98.5%) containing <100 variables (Figure 1). Out of the original 3,285 variables, 28 (0.85%) did not cluster with any other variables. We were able to ascribe a central topic to 384/391 (98.2%) clusters. The degree of relatedness varied widely as discussed below; however, reviewers ascribed some degree of relatedness to 95.1% of applicable clusters in a manual scoring process.

The correlation analysis successfully grouped variables with common themes together. Variable cluster themes included varying aspects of personal health history, family health history, substance use history, dietary intake, and clinical test results. Health history clusters often pertained to specific symptoms or disorders such as history of asthma, history of cardiac surgery, history of high blood pressure. Family health history had similar topics focused on maternal or paternal history (e.g., paternal history of high blood pressure, maternal history of heart attack, and family history of diabetes). Clinical clusters included blood pressure, lipid panel results, and cardiac ultrasound. The cluster themes had varying degrees of breadth. Some were narrow, such as multiple variables capturing marital status, and some were broader, such as a cardiovascular theme that included stroke, heart attack and coronary heart disease.

Inspection of the correlations within the identified clusters reaffirmed known relationships. For example, cluster 7 linked education level, occupation, and level of physical activity at work, and cluster 59 linked a history of stroke with quintessential stroke symptoms (speech/vision problems, dizziness) (Supplemental Table S1). The clustering also matched survey questions focused on human behavior with the corresponding clinical measurements from the subjects. For example, cluster 33 included dietary intake and measured nutrient levels such as variables related to carrot consumption, Vitamin A levels, and carotenoid levels (Supplemental Table S1). While the connection is driven by an underlying biological process (uptake of vitamin A and carotenoids), the variables themselves are dissimilar. Two are measurements of the blood level of nutrients and the third is a dietary intake survey. This was also observed with fish consumption combined with omega3 fatty acid levels and calcium consumption, selenium consumption, and vitamin C consumption linked to their respective blood level measurements.

**FIGURE 2:**
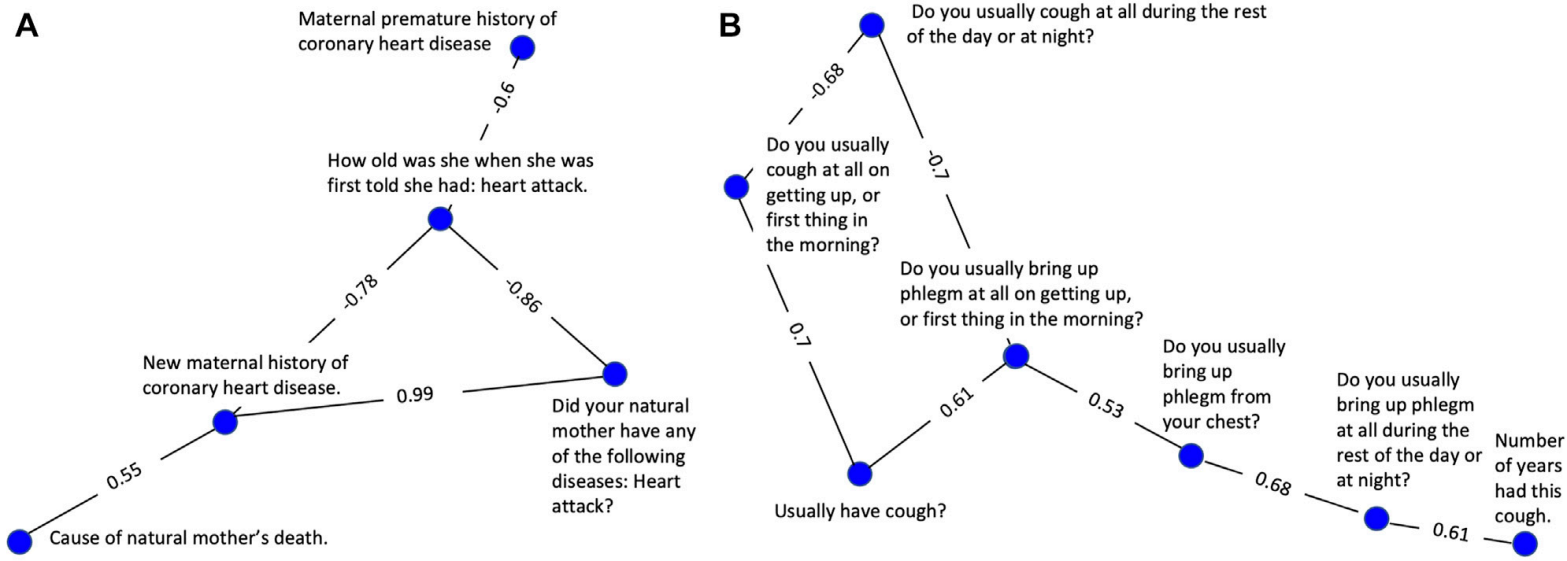
Clusters selected to demonstrate successful clustering by the variable correlation analysis. **(A)** A cluster of variables related to maternal history of heart disease. **(B)** A cluster of variables related to coughing symptoms, frequency, and duration.

In Figure 2A, a strong positive correlation (0.99) was observed between “Did your natural mother ever have any of the following diseases? Heart attack?” and “New maternal history of heart disease,” as anticipated. Respondents answering yes to having a maternal history of heart disease have a considerably higher chance of having a mother who has had a heart attack than respondents answering no. A strong negative correlation between the former variables (−0.86 and −0.78 respectively) and “How old was she [your mother] when she was first told she had: heart attack” was also observed as expected. In fact, this was the case for all categorical variables connected to the continuous age variable in Figure 2A. This is likely because a heart attack at a young age is more prevalent in families with a history of heart disease. This example highlights why the absolute value of the correlation was used during the correlation and clustering process. Strong negative correlations are equally likely to show a meaningful association between two variables as are strong positive correlations.

The cluster represented in Figure 2B consists of 7 highly correlated variables related to the nature and severity of the subject’s cough. A set of 3 variables related to frequency are tightly linked, having edge values from 0.68–0.70. A second set of 3 variables evaluating cough-associated phlegm production were also tightly linked with edge values from 0.53—0.68. An additional variable related to duration was also tightly connected to the group with an edge value of 0.61.

### 3.2 Evaluation of Variable Clusters

**FIGURE 3:**
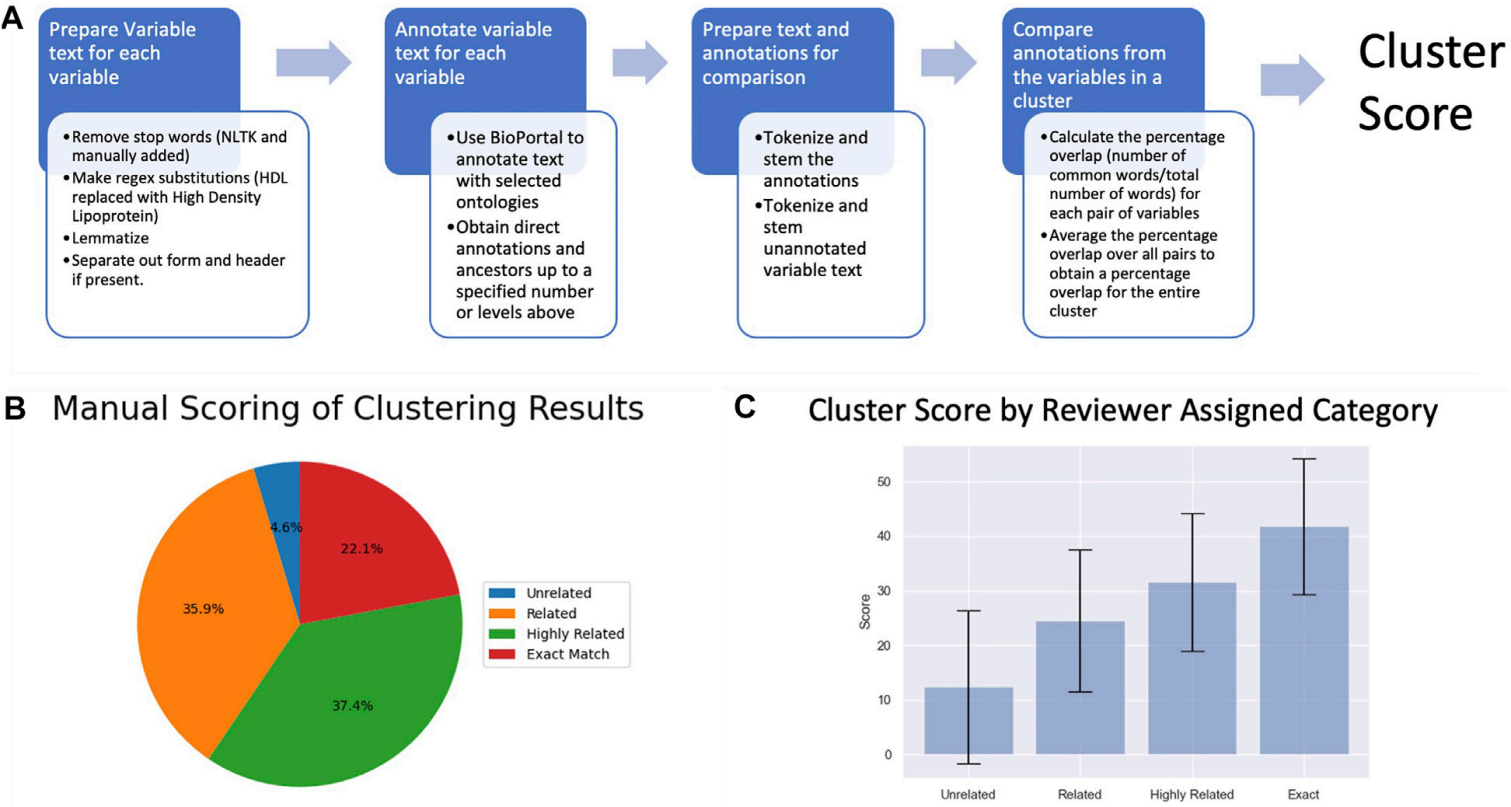
**(A)** A depiction of the NLP-based cluster scoring process. **(B)** Pie chart of the manual scoring of the 391 variable clusters **(C)** Plot of cluster scores for clusters in the different relatedness categories.

We evaluated the clusters using both manual review and NLP of the variable text. Both metrics indicated that the correlation analysis was successful at clustering related variables (Figure 3). The manual evaluation process involved human reviewers categorizing the clusters as Exact, Highly Related, Related, Unrelated or NA (Table 1). In addition to the 391 clusters, 28 out of the 3,285 ARIC variables didn’t cluster with any other variables. Of the 391 clusters, 95.1% were determined by the reviewer to have some degree of relatedness and were classified as Exact, Highly Related, or Related. Only 4.6% were classified as Unrelated (Figure 3B). Examples of clusters in the different relatedness categories can be seen in Table 1. Related clusters contained variables that are loosely related such as variables related to cholesterol, blood pressure, and atherosclerosis. Highly related clusters typically contain orthogonal assays or survey questions that focus on a single endpoint such as how many drinks a person consumes per day or per week combined with how many days a week does the person drink. Exact clusters contain variables such as the same measurement at different time points or survey questions that differ mainly in the terminology used such as being hotheaded vs. having a fiery temper.

Clusters were scored programmatically for relatedness using NLP to compare the text description of the variables. The average score for each category increased by approximately 10 points with increasing relatedness (Figure 3C), corroborating the results from the manual evaluation. A Kruskal-Wallis test found a significant difference in mean between scores for clusters in the different relatedness categories (pvalue = 1.80e-18) further supporting the hypothesis that the calculated relatedness scores reflect the reviewer-assigned categories. Most major discrepancies between the automated scores and human categories occur due to variation in the terminology or phrasing used to convey the same concept. Subtle differences in terminology between variable descriptions which don’t impact a human reviewer’s interpretation (e.g, the presence of a hyphen) can lead to disparate ontology annotations in certain cases.

Clusters 67, 14, and 341 (Table 2) which are related to chronic lung diseases, asthma, and coughing/wheezing respectively, have scores which are congruous with their manual classification. Cluster 67 variables are the same question on different intake forms, cluster 14 variables pertain to different aspects (ever had, age started/stopped, etc.) of the same diagnosis (asthma). Cluster 341 variables encompass symptoms (wheezing/coughing) which are clearly, albeit more loosely, related. Cluster 213 variables are related to diet, specifically baking, but would not be expected to correlate, and thus the cluster was ranked as unrelated.

**TABLE 2:** Examples of clusters which both reflect (67, 14, 341, 213) and do not reflect (70, 49, 403) their programmatically generated scores. The table includes a cluster identifier (Cluster Number), calculated relatedness score from the NLP analysis (Score), relatedness category from manual review (Category), description of the overarching theme of the cluster (Description), and the ARIC variables in the cluster (Variables). Clusters were selected to highlight cases of agreement and disagreement between programmatic scoring and reviewer category assignment. See Supplemental Table S1 for all clusters.

Cluster Number	Score	Category	Description	Variables
67	42.0	Exact	lung health history (lung disease)	Has a doctor ever said that you had any of the following: chronic lung disease, such as chronic bronchitis, or emphysema? Q10g [Home Interview, exam 1]
				[Medical care]. Has a doctor ever said you had any of the following: (read each disease name and code N if No or Never Tested). Q5. Chronic lung disease, such as chronic bronchitis, or emphysema. Q5E [Health/Medical History, exam 2]
14	24.7	Highly Related	lung health history (asthma)	[Asthma]. Ever had asthma? Q35 [Respiratory Symptoms and Physical Activity Form, exam 1]
				[Asthma]. Age asthma started Q37 [Respiratory Symptoms and Physical Activity Form, exam 1]
				[Asthma]. Age asthma stopped. Q39 [Respiratory Symptoms and Physical Activity Form, exam 1]
				[Wheezing]. Age at first attack. Q18 [Respiratory Symptoms and Physical Activity Form, exam 1]
				Has a doctor ever said that you had any of the following: asthma? Q10h [Home Interview, exam 1]
				[Medical care]. Has a doctor ever said you had any of the following: (read each disease name and code N if No or Never Tested). Q5. Asthma. Q5F [Health/Medical History, exam 2]
				[Medical care]. Has a doctor ever said you had any of the following? Asthma. Q6e [Personal History form, exam 4]
				[Asthma]. Still have asthma? Q38 [Respiratory Symptoms and Physical Activity Form, exam 1]
				[Wheezing]. Short Of Breath Wheezing Attack? Q17 [Respiratory Symptoms and Physical Activity Form, exam 1]
341	16.4	Related	lung health history (cough/wheezing)	[Wheezing]. Number years this wheezy or whistling sound been present. Q16 [Respiratory Symptoms and Physical Activity Form, exam 1]
				[Cough]. Number years had trouble with phlegm. Q12 [Respiratory Symptoms and Physical Activity Form, exam 1]
213	7.8	Unrelated	diet	[Other dietary items]. Enter code and specify brand and form below. Q78 [Dietary Intake Form (DTIC), exam 3]
				[Other dietary items]. What kind of fat do you usually use for baking? Q77 [Dietary Intake Form (DTIC), exam 3]
70	6.0	Exact	medication (cholesterol lowering)	Cholesterol lowering medication W/in 2 weeks.: using 2004 Med. code, visit 2 [Cohort, Exam 2]
				Used statin (at visit 2) last 2 weeks (0 = no, 1 = yes) based on 2004 Med. code [Cohort, Exam 2]
49	5.7	Exact	blood pressure measurements (ankle brachial)	Ankle Brachial Index, visit 1, definition 4 [Ankle Brachial Index Data, exam 1]
				Ankle-Brachial index return [Ankle Brachial BP (Blood Pressure—ultrasound work station), exam 1]
403	50.0	Related	medication	[Medication records]. Medication code number. Q12B [Medication Survey Form, exam 2]
				[Medication records]. Medication code number. Q11B [Medication Survey Form, exam 2]

There are also instances where a cluster’s classification and score are incongruous (Table 2). For example, although the cluster 70 variables ask the same question, the scoring algorithm does not recognize the close relationship as the specific wording differs. The phrase “cholesterol lowering medication” does not annotate as a single concept, it is split up into 3 separate concepts whereas “statin” encompasses the entire concept and annotates as “antihyperlipidemic agent” with a parent concept of “Treatment of ischemic stroke” resulting in minimal overlap in the ontology annotations, even including ancestors. Similarly, the discordance between the score (low) and category (Exact) for cluster 49 is likely also due to wording as “ankle brachial index” and “ankle-brachial index” annotate differently. There are also cases where the wording is similar, but the answers would not necessarily be highly related. For example, cluster 403 contains codes for different medications taken by the subject and is ranked as Related. Although the medications may be prescribed for the same or related health issues, they may also be completely unrelated. The wording of the variable, however, is identical except for the question number (“Medication code number. Q12B” and “Medication code number. Q11B”), resulting in a high score.

### 3.3 Grouping Clusters

Communities within the network of clusters were identified and defined as cluster groups. The Louvain algorithm was run after removing edges with weights below a threshold ranging from 0.08 to 0.25. Depending on the threshold, a total of 9–30 cluster groups containing 2 or more clusters were assembled. In addition to these cluster groups the algorithm result in 3–175 clusters that did not group with any other clusters (Supplemental Table S2). The cluster groups ranged in size from 2–44 clusters and the average group size for each pruning threshold ranged from 1.2 to 5.5. This difference in size and number of clusters is expected. Pruning at a lower threshold increases the interconnectivity of the graph, which leads to larger communities. As the threshold goes up, small groups of clusters and single clusters break off from the larger groups, resulting in smaller cluster groups and increasing the number of singleton clusters. The grouping algorithm organized clusters into related groups in many cases although not with the same degree of success as the variable clustering process. There was a mix of cohesive groups with a single overarching theme, larger more disparate groups, and groups consisting of a single cluster. Examples of cluster group themes included general paternal health history, lung health (including emphysema, bronchitis, lung disease clusters), and mental well-being (including clusters related to intrapersonal support and life satisfaction).

**FIGURE 4:**
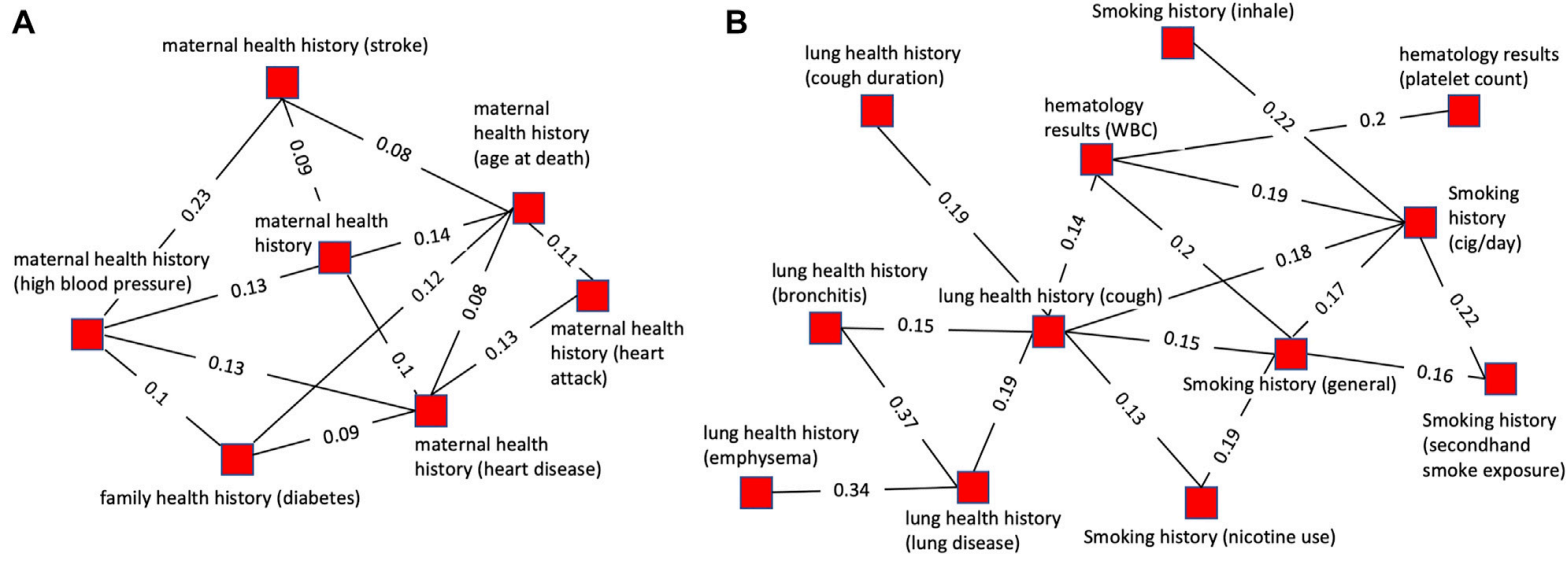
Plots of single cluster groups demonstrating cluster cohesiveness around a central theme. Each node is a variable cluster that is a member of the cluster group, and the group of interconnected nodes is one cluster group. **(A)** An example of a cluster group with clusters relating to maternal health history using a threshold of 0.12 for pruning prior to community finding and 0.07 for viewing. **(B)** An example of a cluster group with clusters relating to cigarette smoking and lung health using a threshold of 0.18 for pruning prior to community finding and 0.12 for viewing.

Figure 4 highlights two cases of the successful grouping of variable clusters into cluster groups. The cluster group shown in Figure 4A consists of 7 clusters all of which are related to maternal health history. Of these, 4 pertain to maternal history of heart disease or health conditions directly related to cardiovascular disease (stroke, heart attack, and blood pressure). One cluster pertains to diabetes which is associated with an increased risk of heart disease. The remaining two clusters are more generic, containing variables related to general maternal health history and mother’s age at death. This cluster group exemplifies a cohesive group of clusters that is unified by an overarching theme, maternal health history.

The cluster group shown in Figure 4B is composed of 12 variables. Of these, 5 variables were related to smoking history, with some specific to certain aspects of smoking history such as secondhand smoke exposure or cigarettes per day. All the other clusters were related to health conditions caused by smoking (lung diseases, emphysema, bronchitis, cough) or factors impacted by smoking (platelets, hematology). This illustrates a grouping of clusters based on underlying biology. The clusters are related to several biologically interdependent themes (smoking, lung health, hematology) as opposed to a single theme as in most of the cluster groups.

**FIGURE 5:**
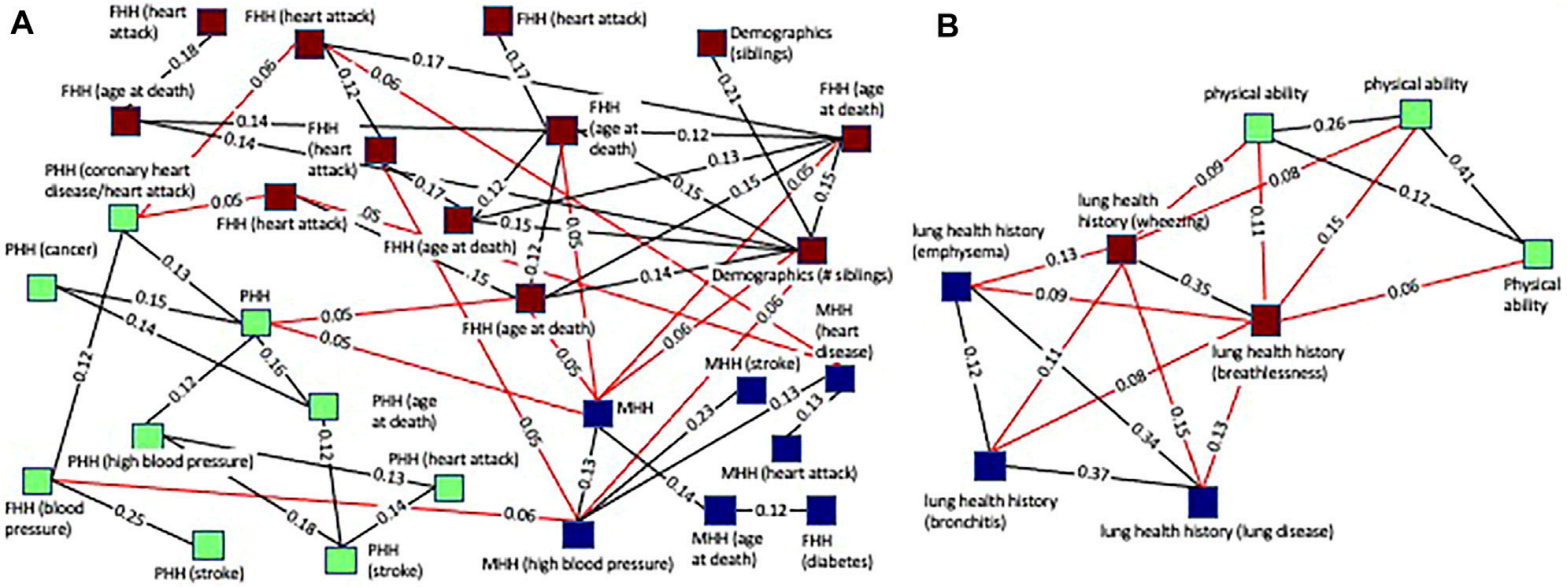
Plots of multiple cluster groups demonstrating interconnectivity between cluster groups. Each node is a variable cluster with cluster groups being identified by node color. Black lines are intra-cluster edges and red lines are inter-cluster edges. The threshold for intra-cluster edges is 0.12 and for inter-cluster edges is 0.05. **(A)** Three interconnected cluster groups related to health history. The green (paternal health history-PHH) and blue (maternal health history-MHH) clusters are linked through the red clusters (family health history-FHH). **(B)** Three interconnected cluster groups. The green (physical activity) and blue (history of lung diseases), cluster groups are linked through the red cluster group (history of wheezing and breathlessness) but not directly connected to each other.

Cluster groups with related themes were more tightly linked and had more inter-group connections than more disparate groups. The interconnectivity of related cluster groups can be seen by plotting multiple cluster groups simultaneously (Figure 5). Figure 5A illustrates the high level of connectivity between nodes in cluster groups related to paternal health history, maternal health history, and family health history. This is driven by connections between family health history and maternal health history as well as family health history and paternal health history. Paternal and maternal health history have a much lower level of interconnectivity and are linked together primarily through each of their close connections with family health history.

Figure 5B illustrates how lung health history and physical ability are connected through three cluster groups with numerous inter-group connections. Similar to the cluster shown in Figure 5A it shows the high level of interconnectivity between cluster groups with themes that are biologically interdependent. Lung health and physical ability are closely linked with each having a direct and robust impact on the other. Notably, the physical ability cluster groups are connected to the lung disease health cluster groups through wheezing and breathlessness, two symptoms of lung disease expected to strongly impact physical ability.

## 4 Discussion

In this study we distilled 14,425 variables from the ARIC study into 391 clusters representing 3,285 variables using a network built from partial correlations among the variables. The clusters were then grouped by calculating inter-cluster distances and using a community finding algorithm on the resultant graph. Hence, the original 14,425 variables were reduced to 9–30 cluster groups (depending on the pruning threshold applied). Whereas 14,425 unordered variables are too numerous for a human reviewer, 3,285 variables grouped into biologically meaningful subsets make human curation feasible. In fact, the manual curation of the 3,285 variables in this study was performed before the programmatic grouping of the clusters suggesting that future iterations of this procedure could be more efficient.

**FIGURE 6:**
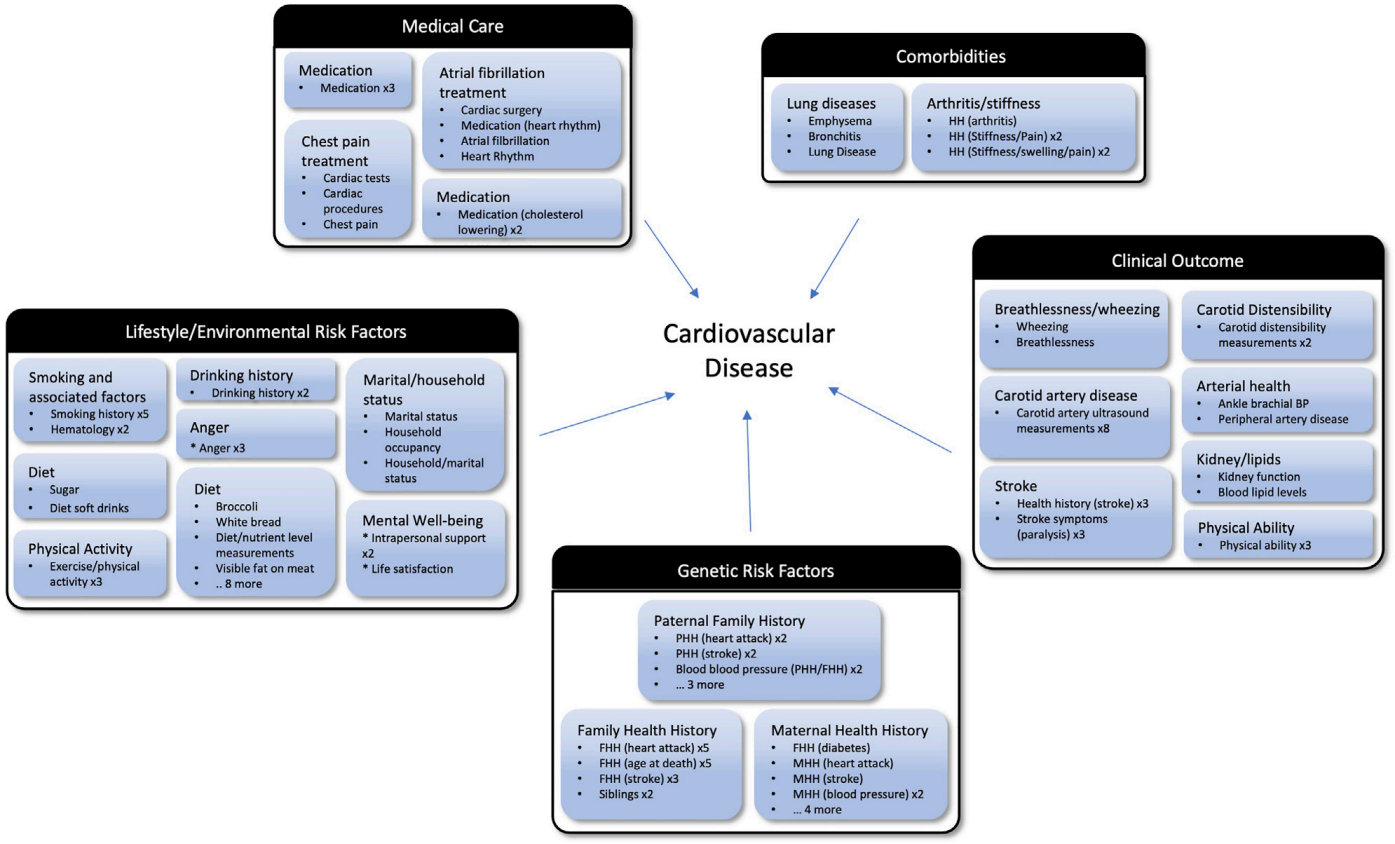
Cluster groups organized into topics based on the goals of the ARIC study. The outer black and white boxes are topics (e.g., Clinical Outcome and Medical Care) and each topic contains multiple cluster groups (e.g., Stroke and Lung Diseases) which are the blue boxes. Listed under the manually assigned label for each cluster groups are bullets representing the clusters which are members of that group. If there are multiple clusters within a group with the same name, after the cluster name they have an “x” and the number of times that cluster appears. For example, Anger x3 means there are three clusters in that group with the name Anger. Abbreviations: MMH, Maternal Health History; PHH, Paternal Health History; MH, Medical History.

Manual curation of the data-driven cluster groups across thresholds identified 5 major topics related to the central goals of the ARIC study, creating a succinct set of topics and cluster groups that captures the fundamental goals of the study (Figure 6). For example, the 3 clusters shown in Figure 5A, would be grouped together in the “Genetic Risk Factors” category. The maternal health history, paternal health history, and family health history cluster groups are all related to family health history and represent the role genetics plays in an individual’s risk of developing cardiovascular disease. If this is the nature component of CVD risk, the nurture component would be the Lifestyle/Environmental Risk Factors topic containing physical activity, smoking, and diet related cluster groups among others. There is some structure to study variables inherent in the study design, so a human curator would never be faced with tens of thousands of unstructured variables. However, many topics are addressed on different surveys, and the laboratory measurements that correspond to certain survey topics are necessarily captured in a different segment of the study data. Our data-driven approach consolidates these variables to make it feasible for a human reviewer to easily capture the key concepts from an epidemiological study. In our proof-of-concept study, fewer than 30 cluster groups with less than 400 clusters were considered. Providing descriptive labels for these groups and clusters can be done by a subject matter expert in a matter of hours compared with weeks to assemble over 10,000 variables *de novo*.

While many important variables are lost when focusing on the 3,285 variables matching our stringent filtering criteria, the themes from the cluster groups support the conclusion that our selection criteria is enriching for highly relevant variables. In fact, the data-driven approach is extracting the variables that are empirically related and may even improve performance by eliminating variables that aren’t performing as expected. In the case where a key variable is missed by our approach, that variable can easily be reincorporated during the human review stage. As shown in Figure 6, the high-level categories identified when reviewing the cluster groups are consistent with the focus of the ARIC study. We see lifestyle, environmental and genetic factors that influence cardiovascular disease. When focusing on the clinical outcomes, we see both phenotypes that lead to atherosclerosis such as high cholesterol as well as diseases resulting from atherosclerosis such as heart failure and stroke. This suggests our approach can facilitate human curation efforts to map variables from one study to another by extracting and organizing the most relevant variables from each study.

Although the variable clusters were cohesive, indicating successful clustering, we did not note any novel correlations among the clusters. This is not surprising because the variables used for this analysis were all selected for a specific purpose as part of the experimental design of the original study in contrast with a more discovery-oriented omics study. The fact that both the original clusters and the subsequent groups of those clusters revealed known associations could be considered a strength of this approach as highlighted above. By grouping similar variables and thereby facilitating the mapping of those variables between studies, our work can enable pooled analyses of larger datasets thereby increasing the power to detect novel GWAS associations. In this manner, the work can indirectly impact novel discoveries. For cohorts that include an omics component such as RNAseq, the correlations between the omics measurements and clusters of related phenotypic variables could be used to guide discovery efforts within a single cohort as well.

This workflow could easily be incorporated into variable mapping efforts like Pan et al. (2012). Each study could be pre-processed to extract the most relevant variables and group those variables thematically. This could also be done in combination with text-based, machine learning approaches that cluster variables based on the similarities in the description of those variables. Text-based approaches have an added advantage of being applicable across studies whereas our workflow is restricted to the measured variables within a single study. However, the two sources of information are orthogonal, as seen when comparing the manual review and NLP scoring of our results, which should make the combination of the two approaches more accurate than either approach individually. In addition, knowledge of the correlation between two variables in one or more studies, could be informative when attempting to estimate a missing variable based on other related variables within a new study.

Limitations with the correlation-based approach are mainly due to the underlying data quality and challenges associated with performing a uniform analysis of all variables from a single study. Data quality issues will also be a problem for downstream analyses, and they are most likely to manifest as unrelated clusters or the presence of an unrelated variable within an otherwise coherent cluster. In both cases, the variables in question can probably be safely discarded from future analyses. Future improvements of our workflow would focus on addressing the latter issue. For example, the distribution of data for certain variables violates the assumptions of the Pearson correlation as do non-linear relationships among the variables. Ideally, each edge weight would be based upon the appropriate association metric, but establishing a fully automated workflow to determine the appropriate metric for each variable pair is a non-trivial exercise. Similarly, a comprehensive review of community finding algorithms for defining the cluster groups was beyond the scope of the current study, but this could potentially improve performance in the future.

Limitations with the NLP methods are primarily driven by the imprecision inherent in written language. The representation of biological concepts via ontologies is maturing, which will greatly improve the results from NLP approaches. In fact, several recent efforts at using NLP and machine learning to predict variable relationships have shown great promise. For example, earlier this year a semantic search tool designed to query biomedical datasets on the variable level using NLP and ontological knowledge graphs (Waldrop et al., 2022), was successfully deployed in the NHLBI’s BioData Catalyst Ecosystem (National Heart, 2020). NLP approaches are also more appropriate to support data harmonization than for applications like AOPs where separate variables related to a common biological event are jointly considered. For example, cluster 33 included both carrot consumption along with Vitamin A and carotenoid levels. While these would never be combined as a pooled study variable, they could all be used a surrogate measures for a key event that includes Vitamin A levels as either a measurable phenotype or a modulating factor.

Using these methods, we have been able to identify biologically meaningful relationships using the underlying data. The resulting variable clusters can then be mapped into knowledge-based systems that model the biological processes underlying disease (Wittwehr et al., 2015; Ankley and Edwards, 2018b; Martens et al., 2018; Slenter et al., 2018; Biomedical Data Translator Consortium, 2019; Watford et al., 2019b; Davis et al., 2019, 2020; Morton et al., 2019; Mortensen et al., 2021). Once mapped to potential disease mechanisms, the data from these existing studies can be modeled in novel ways to create new insights. In cases where the original studies contain an omics component, the variable clusters could be used to assist in discovery-driven analyses of the omics data. As new systems-based models of human disease are developed, these variable clusters should be easily mapped onto those models creating a wealth of data to support those analyses.

Finally, the variable clusters can be mapped onto key events within AOPs describing mechanisms of toxicity. The variables captured in this study would correspond to later events within the AOPs and would include adverse outcomes directly measured in human populations. By mapping NAMs to early key events within the same AOPs, the human data could be used to evaluate the ability of those NAMs to predict toxicity in order to support human health risk assessment decisions. Traditional toxicity data from laboratory animals can be incorporated into the same AOP-based model and help inform the toxicity predictions, but the animal data in this scenario is not intended to take the place of the real-world adverse outcomes that are the target of the risk assessment.

## 5 Conclusion

In conclusion, we present a novel workflow for extracting key variables from a large clinical study and summarizing those variables to enable reuse. This workflow could be incorporated into data harmonization efforts to reduce the human effort required for the initial variable mapping and provide important quantitative information to assist with the harmonization itself. It can also be incorporated into projects focused on organizing knowledge about human disease and the systems biology models built upon those knowledgebases. AOP-based systems models can, in turn, be used to evaluate the predictive value of individual NAMs and to develop integrated models that incorporate data from multiple NAMs as well as traditional animal studies to improve the toxicity predictions.

## Data Availability

Publicly available datasets were analyzed in this study. Data availability and detailed policies for requesting Atherosclerosis Risk in Communities (ARIC) data can be found at https://sites.cscc.unc.edu/aric/pubs-policies-and-forms-pg. Select ARIC data can also be obtained from the NHLBI BioLINCC repository (https://biolincc.nhlbi.nih.gov/home/).
