# Supplementary figures and images for: Correlation Analysis of Variables From the Atherosclerosis Risk in Communities Study
Source: Front Pharmacol. 2022 Jul 11;13:883433. doi: 10.3389/fphar.2022.883433 (PMC9310100; doi:10.3389/fphar.2022.883433)

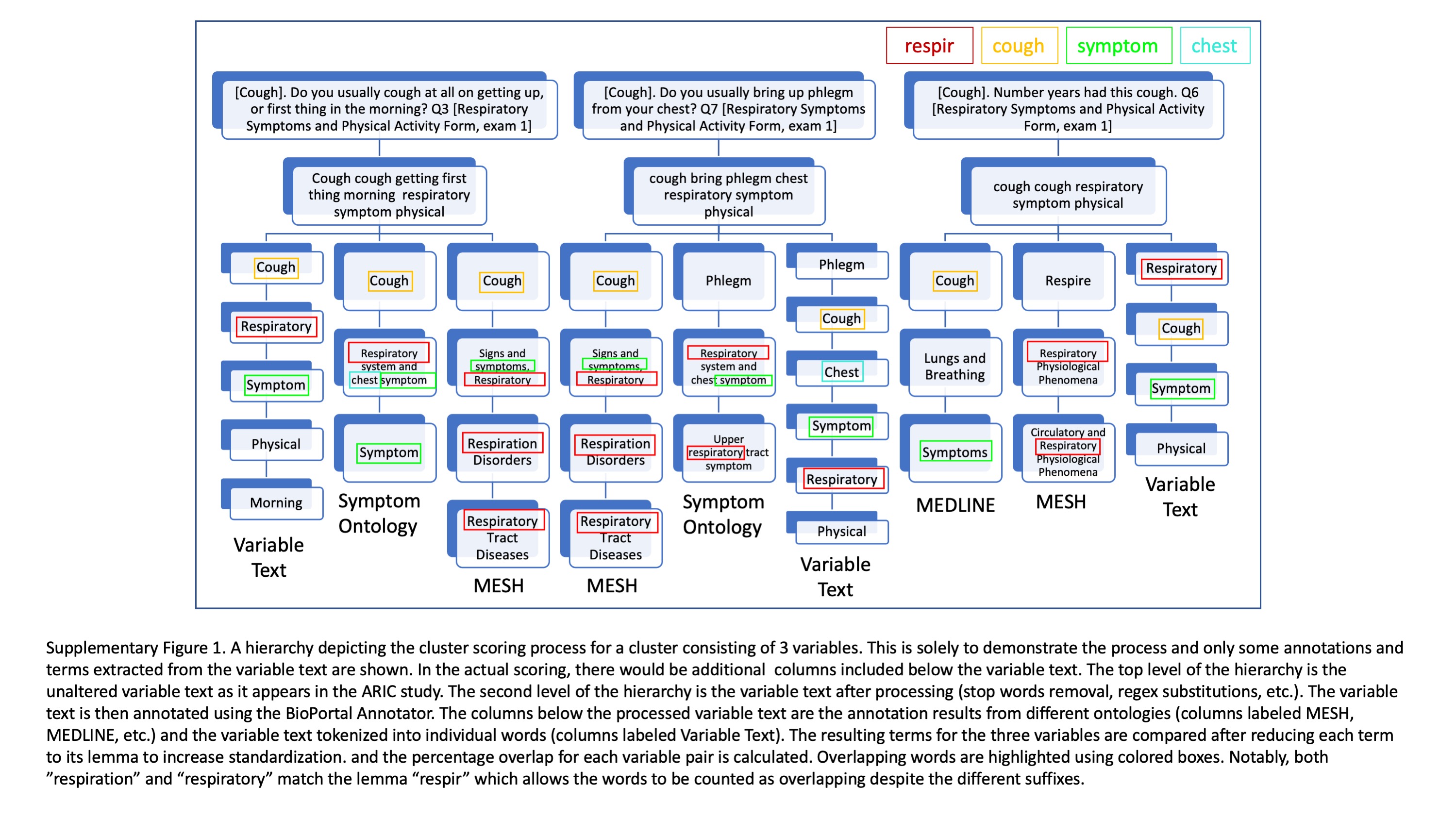

Supplement: Supplementary file 2 [file Image1.JPEG]
